# Endothelium‐related biomarkers and cognitive decline in prevalent hemodialysis patients: A prospective cohort study

**DOI:** 10.1111/ene.16438

**Published:** 2024-08-13

**Authors:** Alexandre Braga Libório, Camila Maroni Marques Freire de Medeiros, Leticia Libório Santos, Luana Silveira de Andrade, Gdayllon Cavalcante Meneses, Alice Maria Costa Martins

**Affiliations:** ^1^ Medical Sciences Postgraduate Program Universidade de Fortaleza‐UNIFOR Fortaleza Ceará Brazil; ^2^ Department of Internal Medicine, Medical School Federal University of Ceará Fortaleza Ceará Brazil; ^3^ Medical Program Universidade de Fortaleza‐UNIFOR Fortaleza Ceará Brazil; ^4^ Medical Sciences Postgraduate Program, Department of Internal Medicine, Medical School Federal University of Ceará Fortaleza Ceará Brazil; ^5^ Clinical and Toxicological Analysis Department, School of Pharmacy Federal University of Ceará Fortaleza Ceará Brazil

**Keywords:** cognition, endothelium, hemodialysis, syndecan‐1

## Abstract

**Introduction:**

Cognitive decline is prevalent in maintenance hemodialysis patients. The blood–brain barrier has been implicated in cognitive decline. In this prospective cohort study, we investigated the associations between endothelium‐related biomarkers and steeper cognitive decline in this population.

**Methods:**

Cognitive function was assessed using the Portuguese‐adapted Cambridge Cognitive Examination (CAMCOG) with items of the Mini‐Mental State Examination (MMSE). Endothelium‐related biomarkers included syndecan‐1, ICAM‐1, VCAM‐1 and angiopoietin‐2 (AGPT2). Patients were followed up for 4 years, and cognitive assessments were repeated. Multinomial regression analyses were performed to evaluate associations between biomarkers and cognitive decline.

**Results:**

A total of 216 patients completed the test battery at baseline. After 4 years, 102 patients had follow‐up data. There was a significant decrease in cognitive function according to the CAMCOG and MMSE scores: a change of −0.39 (95% CI −0.27 to −0.51) and −0.51 (95% CI −0.27 to −0.76) standard deviation (SD) of the baseline scores. Additionally, executive function but not memory significantly decreased. Syndecan‐1 level was independently associated with steeper cognitive decline; each increase in the SD of the syndecan‐1 level was associated with a decrease in the CAMCOG of 0.20 (95% CI 0.07–0.33) SD from baseline. Syndecan‐1 was associated with a steeper decline in MMSE score (*β* 0.54, 95% CI 0.28–0.81) and executive function (*β* 0.17, 95% CI 0.02–0.32). Syndecan‐1 predicted severe cognitive impairment with an area under the curve for receiver operating characteristic curves of 0.75 (95% CI 0.64–0.83).

**Conclusion:**

Our findings highlight the potential of syndecan‐1, a biomarker of endothelium glycocalyx derangement, as a predictor of steeper cognitive decline in prevalent hemodialysis patients.

## INTRODUCTION

Cognitive impairment is common and frequently severe in patients on maintenance hemodialysis. The prevalence of moderate cognitive impairment is 30%–60%, which is at least twice as high as that in the general population [1, 2, 3]. Notably, cognitive impairment contributes to increased morbidity and mortality [3, 4]. Cognitive impairment is not a statistical process and, over time, hemodialysis patients continue to experience significant cognitive decline [5, 6].

Although the prevalence of certain risk factors for cognitive impairment (mainly vascular dementia), such as advanced age [7], high blood pressure [8], diabetes mellitus [9] and dyslipidemia [10], is high in hemodialysis patients, the prevalence of these risk factors alone does not explain the frequency or severity of cognitive impairment in this population. Specific risk factors in hemodialysis patients, such as metabolic acidosis [11] and acute decline in cerebral blood flow [12, 13, 14, 15], have been suggested to contribute to cognitive decline; however, these risk factors must be studied further. Despite all these risk factors, in a prospective study of hemodialysis patients only age was associated with greater cognitive decline [7].

There are several lines of evidence supporting a prominent role for vascular factors in cognitive impairment, and small‐vessel cerebrovascular disease has been estimated to contribute to cognitive impairment in hemodialysis patients [16]. Moreover, disruption of the blood–brain barrier appears to be an important mechanism in neurodegenerative pathologies [17]. By evaluating the regional blood–brain barrier, Nation et al. showed that individuals with early cognitive dysfunction develop brain capillary damage and blood–brain barrier breakdown [18]. In animal models, uremic toxins such as indoxyl sulfate led to blood–brain barrier disruption associated with cognitive impairment [19].

At the capillary level, the endothelium is an important component of the blood–brain barrier, and endothelial dysfunction can be associated with cognitive impairment [20]. Among the endothelium‐related biomarkers, four have different endothelial functions/structures: intercellular adhesion molecule‐1 (ICAM‐1) [21] and vascular cell adhesion protein‐1 (VCAM‐1) [22], which are related to endothelial cell activation, and angiopoietin‐2 (AGPT2) [23], an endothelial growth factor that promotes polymorphonuclear cell infiltration and induces endothelial cell apoptosis. Finally, syndecan‐1 is a marker of endothelial glycocalyx derangement [24].

In the present study we hypothesized that endothelium‐related biomarkers can predict cognitive decline in prevalent hemodialysis patients.

## METHODS

### Patient selection

The complete inclusion and exclusion criteria were previously described [25]. Briefly, we initially selected 267 adult patients who were under maintenance hemodialysis for at least 3 months with Portuguese language fluency and sufficient visual and hearing acuity to complete the neurocognitive test. The exclusion criteria included the absence of acute or chronic psychosis or dementia, acute illness at the time of the assessment and a life expectancy <6 months or anticipated kidney transplantation. The study was approved by an institutional review board (Ethical Committee of Universidade Federal do Ceará), and all participants gave free and informed consent before the cognitive tests were performed.

### Demographic and clinical data

Demographic, clinical and laboratory factors were ascertained at the time of the cognitive testing. Demographic data (age, sex and ethnicity) were obtained from the participants' self‐reports, medical charts and dialysis facility databases. Education (years of formal education) was assessed using the patient questionnaire. Medical history, including history of cardiovascular disease, diabetes, hypertension and smoking (previous or actual), was defined by patient history or documentation in the patient's electronic chart. The dialysis vintage was obtained from the dialysis facilities' electronic records. Pre‐dialysis blood tests included serum albumin, hemoglobin, calcium and phosphorus. The single‐pool *K*_*t*_/*V* was calculated using the pre‐ and post‐dialysis serum urea levels.

### Biomarker measurement

As previously described [25], syndecan‐1 was measured as a biomarker of endothelial glycocalyx injury (Abcam, Cambridge, MA, USA). The intra‐assay coefficient of variation was 6.2%. ICAM‐1, a marker of endothelial cell activation, was measured using a commercially available enzyme‐linked immunosorbent assay (ELISA) kit (Life Technologies Brasil, São Paulo, Brazil), with an intra‐assay coefficient of 8.4%. Additionally, VCAM‐1 was measured using a commercially available ELISA kit (Abcam, Cambridge, UK), with an intra‐assay coefficient of 5.9%. Angiopoietin‐2 was measured using an ELISA (R&D Systems, Minneapolis, MN, USA). The intra‐assay coefficient of variation was 5.3%. All measurements were performed in duplicate.

### Cognitive assessment

Cognitive function was assessed using the Portuguese‐adapted and validated Cambridge Examination for Mental Disorders of the Elderly (CAMDEX) [26, 27]. The CAMDEX contains the Cambridge Cognitive Examination (CAMCOG) [26], a 60‐item scale divided into 11 domains: orientation, language (comprehension and expression), memory (remote, recent and learning), attention, praxis, abstract thinking, perception and calculation. The maximum possible score is 105. The memory subscore was derived from the combined scores for remote, recent and learning memory, with a maximum possible score of 27. The executive function subscore combined scores for fluency of expression, ideation, abstract thinking and visual reasoning with a maximum possible score of 28. All items of the Mini‐Mental State Examination (MMSE) [28], a standardized test assessing cognitive and mental states that is useful for screening, are also incorporated into the CAMDEX.

The CAMDEX was applied by research assistants after training and direct observation by the study psychiatrist (C.M.M.F.M.). To maintain quality and interrater reliability, the tests were performed by the study psychiatrist. To limit participant fatigue, all testing was completed during the first hour of hemodialysis or before the start of a dialysis session.

### Follow‐up

Patients were followed up for 4 years. We obtained the survival status of all patients through periodic electronic medical record monitoring, as well as from each patient's dialysis unit. Similarly, we collected information on censoring events such as kidney transplantation, dialysis modality changes, and transfer to outside the dialysis unit. The follow‐up time was defined as the time elapsed from the initial CAMCOG test. The consenting participants underwent a repeat cognitive assessment at the 4‐year follow‐up. The maximum allowable temporal deviation was 3 months beyond the 4‐year follow‐up period. Patients who experienced stroke with severe language fluency or sufficient visual or hearing acuity during follow‐up were excluded.

### Outcomes

The primary outcome was the change in the CAMCOG score after 4 years. The secondary outcomes included changes in the MMSE score and the memory and executive function subscores. Additionally, based on a previous study in a Brazilian population [29], we defined a CAMCOG score at follow‐up <69 to test the discriminatory capacity of the syndecan‐1 level for predicting severe cognitive impairment.

### Statistical analysis

Descriptive statistics were generated for baseline characteristics and are reported as frequency counts and proportions for categorical and binary variables, means ± standard deviations (SDs) for continuous normally distributed variables, and medians with interquartile ranges (IQRs) for skewed variables.

For better understanding and comparison, changes in CAMCOG, MMSE, memory, and executive subscores are presented as both the mean change in score and as standardized to SD from the baseline score as specified.

For linear regressions, endothelium‐related biomarker values were standardized, and changes in cognitive scores were transformed into SD changes from baseline values. Variables were analyzed prior to excluding nonlinear associations with the outcomes. Simple linear regressions were performed for the effect of each endothelium‐related biomarker on the primary and secondary cognitive measurements. In the adjusted models, we included variables associated with cognitive decline in simple linear regression and variables expected to be associated with cognitive decline from previous studies, namely age, cardiovascular disease and diabetes mellitus. Additionally, we adjusted for years of education. Residual plots (histograms, quantile–quantile, and residuals vs. expected values) were assessed to evaluate the models' fits to their underlying assumptions in multiple linear regression.

We constructed scatter plots of each participant's observed CAMCOG score according to baseline syndecan‐1 measurements at each time point to visualize the differences between baseline and follow‐up status, as well as overall changes in the cognitive measures with time. Adjusted linear mixed model regression lines with 95% confidence intervals (95% CIs) were fitted.

Additionally, the area under the curve (AUC) for receiver operating characteristic (ROC) curves was calculated for syndecan‐1 to predict severe cognitive impairment at follow‐up. All the statistical analyses were performed using R Studio (version 3.6.5; R Foundation for Statistical Computing, Vienna, Austria) and SPSS (version 20.0; IBM, Armonk, NY, USA).

## RESULTS

### Patient characteristics

**FIGURE 1:**
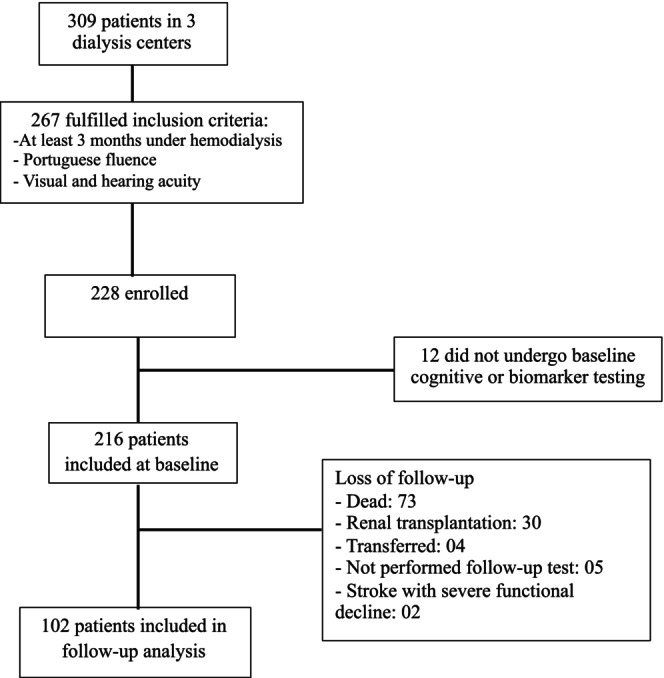
Flow diagram describing the disposition of patients enrolled in the study.

As previously reported, of 267 patients who were eligible for inclusion in the cohort, 216 (94.7%) completed cognitive testing, and blood samples were collected at baseline. During the 4‐year follow‐up, 73 (33.8%) patients died, 30 (13.9%) underwent renal transplantation, 4 (1.9%) were transferred to another clinical facility, 5 (2.3%) did not complete the follow‐up cognitive tests and 2 (0.9%) had stroke with severe and rapid functional decline. Endothelium‐related biomarker levels were similar between those with or without complete follow‐up, and there was a trend toward higher CAMCOG scores in those with complete follow‐up (Table S1). Overall, 102 patients remained in the final analysis of the present longitudinal cohort; see Figure 1 for a complete flowchart of the patients.

The mean age was 49.8 ± 14.4 years and 61 (59.8%) patients were male. At the beginning of the cohort, the median duration of dialysis was 36 (IQR 13–93) months. The majority of patients had arterial hypertension (*n* = 82, 80.4%), 37 (26.2%) had diabetes mellitus, 16 (15.7%) had diagnosed cardiovascular disease and 2 had previous stroke. The median number of years of schooling was 12 (IQR 5–12) and 34 (33.3%) patients had a previous or actual smoking history. The complete baseline cohort characteristics and laboratory values are shown in Table 1.

**TABLE 1:** Baseline characteristics of the study sample.

Characteristic	Value
Age (years), mean ± SD	50.3 ± 13.7
Male, *n* (%)	61 (59.8)
Years of education, median (IQR)	12 (5–12)
Hypertension, *n* (%)	82 (80.4)
Diabetes mellitus, *n* (%)	37 (36.3)
Cardiovascular disease, *n* (%)	16 (15.7)
Past or current smoking, *n* (%)	34 (33.3)
Dialysis vintage (months), median (IQR)	36 (13–93)
Single‐pool *K*_*t*_/*V*, mean ± SD	1.6 ± 0.4
Hemoglobin (g/dL), mean ± SD	11.6 ± 1.7
Phosphorus (mg/dL), mean ± SD	5.4 ± 1.4
Albumin (g/dL), mean ± SD	4.1 ± 0.5
Parathormone (pg/mL), median (IQR)	156 (93–330)
Angiopoietin‐2 (ng/mL), median (IQR)	1.1 (0.7–1.9)
ICAM‐1 (ng/mL), median (IQR)	192 (144–258)
VCAM‐1 (ng/mL), median (IQR)	1377 (1148–1555)
Syndecan‐1 (ng/mL), median (IQR)	159 (98–251)

Abbreviations: ICAM, intercellular adhesion molecule; IQR, interquartile range; SD, standard deviation; VCAM, vascular cell adhesion molecule.

At baseline, the median CAMCOG and MMSE scores were 85 (IQR 75–91) and 26 (IQR 23–29), respectively. The scores for the memory and executive domains of the CAMCOG were 20 (IQR 17–22) and 19 (IQR 16–22), respectively.

### Endothelium‐related biomarkers and baseline cognitive function

Data about correlations between all included patients at baseline (*n* = 216) were published previously [25]. Considering that only 102 patients completed the follow‐up, we could not detect any significant associations of endothelium‐related biomarkers (namely angiopoietin‐2, ICMA‐1, VCAM‐1 and syndecan‐1) with cognitive function parameters (CAMCOG or MMSE) at baseline (Table S2).

### Cognitive decline

**FIGURE 2:**
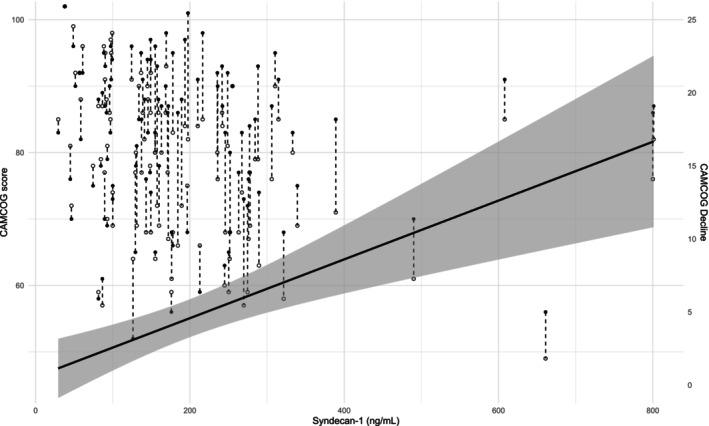
Comparisons of Cambridge Cognitive Examination (CAMCOG) scores at baseline and at the 4‐year follow‐up. Scatter plots displaying individual participant data as a function of syndecan‐1 (baseline, filled circle; follow‐up, empty circle). Dashed lines represent individual participant trajectories over time. The slopes of the solid lines and 95% confidence intervals represent the adjusted overall effect of syndecan‐1 on the differences in the baseline and 4‐year follow‐up CAMCOG scores.

There was a significant decrease in the CAMCOG (change of −4.5 [95% CI −5.9 to −3.1] score or − 0.39 [95% CI −0.27 to −0.51] SD of baseline) and MMSE (change of −1.9 [95% CI −2.9 to −1.0] score or −0.51 [95% CI −0.27 to −0.76] SD of baseline) scores. Figure 2 shows the comparison of CAMCOG scores at baseline and 4 years later. In relation to the memory and executive subscores, there was a significant decline only in the last subscore: a change of −0.1 (95% CI −0.8–0.6) in the baseline score or 0.02 (95% CI −0.16–0.19) in the baseline score and a change of −1.4 (95% CI −2.0 to −0.8) or −0.36 (95% CI 0.22 to –0.50) SD in the baseline score, respectively. With the exception of those domains related to memory (remote, recent and learning), perception and attention significantly decreased in almost all CAMCOG subsets (Table S3).

### Endothelium‐related biomarkers and cognitive decline

According to the univariate analysis, age and syndecan‐1 were associated with a steeper CAMCOG decline, with a *β* coefficient of 0.11 (95% CI 0.02–0.19) for the decrease in the baseline score per year of follow‐up for every 10 additional years of age and 0.24 (95% CI 0.13–0.35) for the baseline score for each SD increase in the syndecan‐1 level. No significant associations were detected for angiopoietin‐2, ICAM‐1 or VCAM‐1. In relation to MMSE and executive function decline, only age and syndecan‐1 remained significantly associated with a decrease in both (see Table 2 for the *β* coefficients of endothelium‐related biomarkers and CAMCOG, MMSE and executive function declines). No significant association with cognitive decline according to the CAMCOG score was observed for comorbidities (arterial hypertension, diabetes mellitus or cardiovascular disease), hemodialysis vintage or laboratory parameters, and years of education tended to be associated with a shallower cognitive decline (Table S4).

**TABLE 2:** Effect of baseline endothelium‐related biomarkers on the slope (faster decline) of cognitive scores.

	CAMCOG		Executive function		MMSE	
Biomarker	Unadjusted	Adjusted	Unadjusted	Adjusted	Unadjusted	Adjusted
Angiopoietin‐2	0.11 (−0.01–0.23)*	0.06 (−0.08–0.18)	0.08 (−0.07–0.22)	0.01 (−0.14–0.17)	0.02 (−0.23–0.28)	−0.02 (−0.30–0.25)
ICAM‐1	−0.07 (−0.19–0.05)	−0.06 (−0.18–0.06)	−0.06 (−0.21–0.08)	−0.05 (−0.19–0.09)	−0.03 (−0.16–0.07	−0.03 (0.09–0.08)
VCAM‐1	0.02 (−0.10–0.14)	0.01 (−0.12–0.13)	0.09 (−0.05–0.24)	0.11 (−0.03–0.25)	−0.02 (−0.27–0.24)	0.01 (−0.26–0.26)
Syndecan‐1	0.24 (0.13–0.35	0.20 (0.07–0.33)	0.24 (0.11–0.38)	0.17 (0.02–0.32)	0.46 (0.23–0.70)	0.54 (0.28–0.81)

*Notes:* For easy interpretation and comparison, endothelium‐related biomarkers were analyzed as standardized values and cognitive scores were analyzed as standardized changes from baseline. For example, for each increment of 1 standard deviation (SD) of syndecan‐1, the CAMCOG score exhibited a steeper decline of 0.20 SD from baseline values in the adjusted analysis. Adjusted for age, years of education, diabetes mellitus and cardiovascular disease. Abbreviations: CAMCOG, Cambridge Cognitive Examination; ICAM, intercellular adhesion molecule; MMSE, Mini‐Mental State Examination; VCAM, vascular cell adhesion molecule.

* *p* = 0.07.

In the multivariate analysis, after adjusting for age, years of education and other variables already known to be associated with cognitive decline in hemodialysis patients (cardiovascular disease and diabetes mellitus) (see Methods), syndecan‐1 remained associated with steeper cognitive decline. Figure 2 shows the adjusted linear regression line between syndecan‐1 and CAMCOG decline with 95% CI. Each increase in the SD of the syndecan‐1 level was associated with a decrease in the CAMCOG score of 0.20 (95% CI 0.07–0.33) SD from the baseline score – the *β* coefficient. The results were similar for the MMSE score (*β* coefficient 0.54, 95% CI 0.28–0.81) and executive function (*β* coefficient 0.17, 95% CI 0.02–0.32).

### Syndecan‐1 as a predictor of severe cognitive decline

**FIGURE 3:**
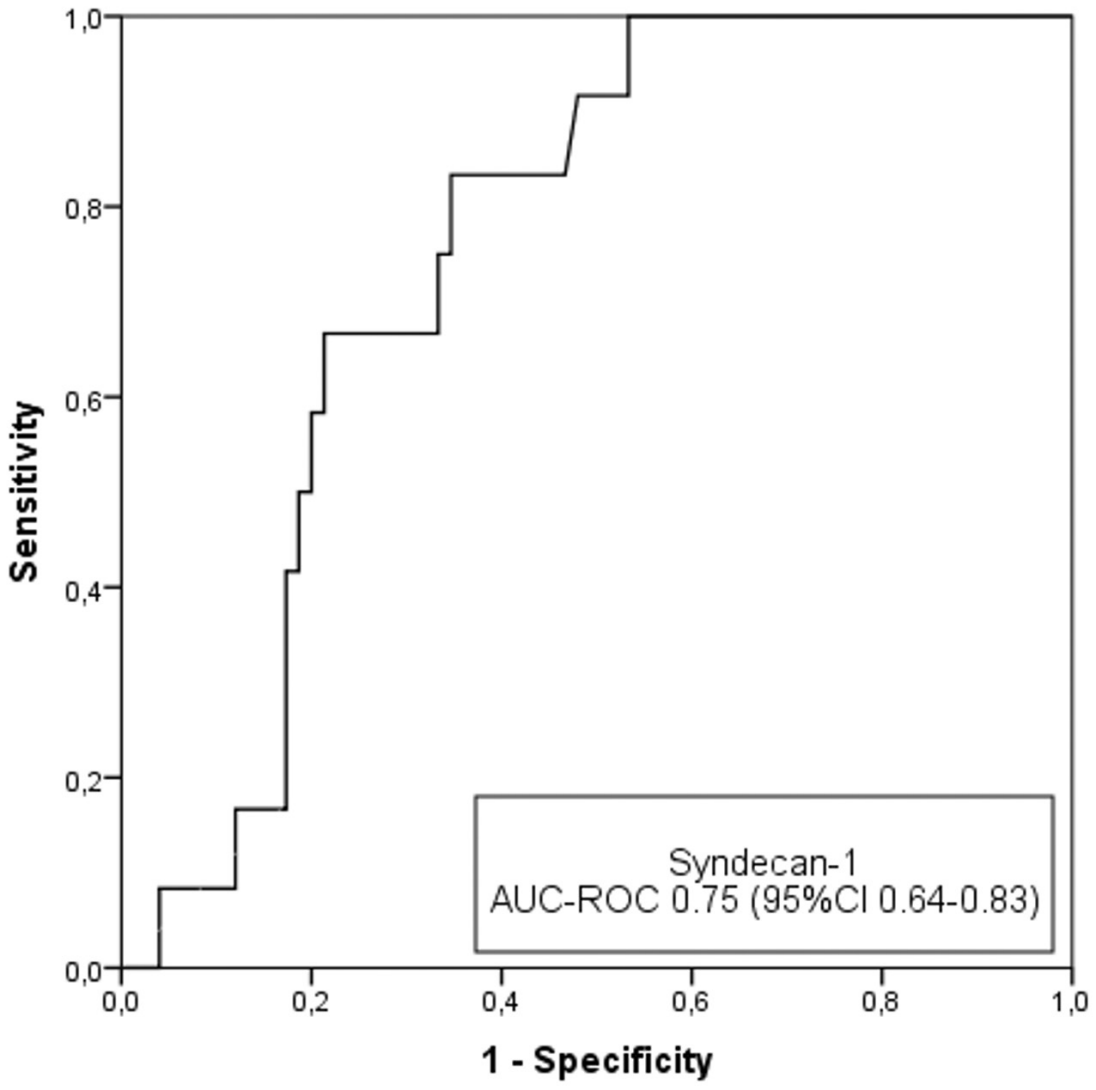
Diagnostic performance of the syndecan‐1 value for the detection of severe cognitive impairment (Cambridge Cognitive Examination [CAMCOG] score <69). AUC‐ROC, area under the curve for receiver operating characteristic curves.

We also evaluated whether syndecan‐1 can be used as a predictor of severe cognitive impairment. This cutoff point (CAMCOG score <69) was defined in the Methods section. At baseline, 86 patients had CAMCOG scores >69, and 17 (19.8%) of these patients developed severe cognitive impairment. Syndecan‐1 had good discriminatory capacity, with an AUC‐ROC of 0.75 (95% CI 0.64–0.83) (Figure 3).

## DISCUSSION

In the present study, we evaluated a cohort of patients undergoing maintenance hemodialysis with a 4‐year follow‐up and identified syndecan‐1, a biomarker of endothelial glycocalyx damage that is independently associated with steeper cognitive decline according to the CAMCOG and MMSE. Additionally, we showed that a decrease in executive function was associated with syndecan‐1. Moreover, the syndecan‐1 level had good discriminative capacity for predicting severe cognitive impairment.

Our study included a 4‐year follow‐up period during which we observed a significant cognitive decline. Drew et al., in their evaluation of a cohort comprising prevalent hemodialysis patients over a median duration of 2 years, reported a cognitive decline rate of −0.09 SD per year [7]. Remarkably, our findings closely mirror theirs, with a comparable reduction of −4.5 SD over 4 years in our cohort. Notably, akin to Drew et al.'s observations [7], only memory function exhibited a nonsignificant decline, while advancing age emerged as a robust predictor of cognitive deterioration. This comparative analysis assumes significance, as it underscores the consistency of our results with those obtained in American cohorts, despite our study's focus exclusively on Brazilian patients.

Numerous studies have linked various changes in vascular function to cognitive decline in both chronic kidney disease patients and hemodialysis patients [30, 31, 32, 33]. Indeed, previously published baseline findings from our current cohort have shown associations between syndecan‐1 and angiopoietin‐2 and cognition [25]. However, few studies have prospectively evaluated the role of endothelium‐related biomarkers in cognitive decline [34, 35]. In a large prospective study, Holm et al. [35] demonstrated that a precursor of atrial natriuretic peptide, a regulator of endothelial function, serves as an independent predictor of all‐cause and vascular dementia in the general population. To the best of our knowledge, our study represents the first prospective investigation evaluating the role of an endothelium‐related biomarker in cognitive decline among hemodialysis patients.

The pathophysiological relationship between the increase in syndecan‐1 expression and cognitive decline has led to several hypotheses. Considering that some studies suggest that syndecan‐1 expression is reduced or absent in brain endothelial cells [36, 37] but is present in the brainstem [37], it can be speculated that the increased syndecan‐1 might originate from this area. However, it appears unlikely that this amount is sufficient to cause a significant increase in serum syndecan‐1 levels. Another hypothesis is that higher syndecan‐1 levels reflect systemic endothelial glycocalyx damage. Although cerebral vessels do not express syndecan‐1, they are exposed to various damaging factors (systemic arterial hypertension, hemodialysis‐related hypoperfusion [32] and others), such as systemic circulation, so syndecan‐1 can reflect systemic endothelial glycocalyx damage, including cerebral vessel damage. As partly explained in the Introduction, uremic toxins appear to damage the blood–brain barrier [19, 38], and the endothelial glycocalyx is an important component of this barrier [39]. Therefore, the exposure of endothelial cells to uremic toxins is increased. Whether our results can be applied to the general population remains an area that warrants further research.

We also assessed whether syndecan‐1 could predict the development of severe cognitive impairment. To define severe cognitive impairment, we used a CAMCOG cutoff score of 69, which was associated with dementia in a Brazilian study with a similar education level [29]. This score is lower than that previously reported in other populations; in the Dutch and British populations [26, 40], the CAMCOG score that best discriminates dementia was 79/80. This difference indicates that studies need to be conducted in other populations to ensure the external validity of our findings; however, syndecan‐1 is a candidate for use in the stratification of hemodialysis patients for developing dementia.

Our study has several strengths. First, this study contributes to a limited body of prospective studies evaluating cognitive decline in hemodialysis patients. Additionally, we employed the CAMCOG score to assess cognition, an extensive test offering advantages over brief screening tools by comprehensively covering various cognitive functions within a relatively brief duration. Moreover, the CAMCOG is proficient in detecting mild cognitive deterioration and minimizes ceiling effects [26]. Finally, we decided to utilize only outcomes demonstrating clinical significance, such as CAMCOG and MMSE total scores, along with executive/memory subscores, thereby avoiding multiple analyses across various CAMCOG domains. The results remained consistent across these selected outcomes.

We acknowledge several important limitations of our study. As discussed earlier, the study was conducted in only one developing country, potentially limiting the generalizability of the findings. Additionally, we included only prevalent hemodialysis patients. While the possibility of survival bias exists because our cohort consisted exclusively of prevalent hemodialysis patients, the inclusion of incident hemodialysis patients could introduce other biases because these patients may have worse cognitive function due to uremia, acute illness and concurrent hospitalization [41]. Finally, the high mortality rate observed after 4 years in our study is comparable to that of other patients in maintenance HD cohorts [7], but it could be associated with bias due to loss to follow‐up and periodic assessment of cognitive function could minimize the loss of information. However, we did not find any significant difference in syndecan‐1 levels or baseline cognitive function between patients who completed follow‐up and those who were lost to follow‐up, reducing the chance of follow‐up bias.

In conclusion, within a prospective cohort of hemodialysis patients, syndecan‐1, a biomarker indicative of endothelial glycocalyx derangement, exhibited a significant correlation with accelerated cognitive decline, as assessed by the CAMCOG and MMSE. Furthermore, it demonstrated a parallel association with heightened deterioration in executive function. Additionally, within this demographic, syndecan‐1 displayed promising discriminative ability in predicting severe cognitive impairment.

## AUTHOR CONTRIBUTIONS

**Alexandre Braga Libório:** Conceptualization; writing – original draft; methodology; formal analysis. **Camila Maroni Marques Freire de Medeiros:** Conceptualization; investigation; writing – review and editing. **Leticia Libório Santos:** Writing – review and editing; investigation. **Luana Silveira de Andrade:** Investigation; writing – review and editing. **Gdayllon Cavalcante Meneses:** Investigation; writing – review and editing. **Alice Maria Costa Martins:** Supervision; writing – review and editing.

## FUNDING INFORMATION

This work was supported by the Conselho Nacional de Desenvolvimento Científico e Tecnológico (Grant No.: 306377/2022‐5).

## CONFLICT OF INTEREST STATEMENT

The authors declare no competing interests.

## ETHICS STATEMENT

Informed consent was obtained from each participant and the study was approved by an institutional review board, the Ethical Committee of Universidade Federal do Ceará.

## CONSENT

This is available on request from the corresponding author.

## Supporting information


Table S1.

Table S2.

Table S3.

Table S4.

## Data Availability

The anonymized data presented in this article are available at the request of a qualified investigator, after review by the corresponding author.
