# Supplementary material for: Endothelium‐related biomarkers and cognitive decline in prevalent hemodialysis patients: A prospective cohort study
Source: Eur J Neurol. 2024 Aug 13;31(12):e16438. doi: 10.1111/ene.16438 (PMC11555003; doi:10.1111/ene.16438)
Supplement: Supplementary file 1 — Table S1. [file ENE-31-e16438-s004.docx]

**Supplementary Table S1: Baseline endothelium-related biomarkers and CAMCOG score in patients according to follow-up.**

|  | **Lost follow-up (n=114)** | **Complete follow-up (n=102)** | **p** |
| --- | --- | --- | --- |
| **Angiopoietin-2 (ng/mL), median (IQR)** | 1.3 (0.8-2.0) | 1.1 (0.7-1.9) | 0.11 |
| **ICAM-1 (ng/mL), median (IQR)** | 233 (155-384) | 192 (144-258) | 0.67 |
| **VCAM-1 (ng/mL), median (IQR)** | 1,409 (1,143-1,593) | 1,377 (1,148-1,555) | 0.74 |
| **Syndecan-1 (ng/mL), median (IQR)** | 128 (99-222) | 159 (98-251) | 0.51 |
| **CAMCOG score, median (IQR)** | 82 (66-94) | 85 (75-91) | 0.22 |
