# Supplementary material for: Endothelium‐related biomarkers and cognitive decline in prevalent hemodialysis patients: A prospective cohort study
Source: Eur J Neurol. 2024 Aug 13;31(12):e16438. doi: 10.1111/ene.16438 (PMC11555003; doi:10.1111/ene.16438)
Supplement: Supplementary file 2 — Table S2. [file ENE-31-e16438-s003.docx]

|  | CAMCOG | MMSE |
| --- | --- | --- |
| Angiopoietin-2 | 0.070 | 0.025 |
| ICAM-1 | -0.079 | -0.067 |
| VCAM-1 | -0.021 | 0.078 |
| Syndecan-1 | -0.092 | 0.105 |

**Supplementary Table S2:** Endothelial-related biomarker and CAMCOG/MMSE Spearman correlations at baseline.

VCAM-1: vascular cell adhesion protein 1; ICAM-1: intercellular adhesion molecule-1; CAMCOG: Cambridge Cognitive Examination MMSE: Mini-Mental State Examination.
