# Supplementary material for: Endothelium‐related biomarkers and cognitive decline in prevalent hemodialysis patients: A prospective cohort study
Source: Eur J Neurol. 2024 Aug 13;31(12):e16438. doi: 10.1111/ene.16438 (PMC11555003; doi:10.1111/ene.16438)
Supplement: Supplementary file 3 — Table S3. [file ENE-31-e16438-s001.docx]

**Supplementary Table S3:** Mean change from baseline to the end of follow-up in each CAMCOG domain.

| **CAMCOG subscore** | **Mean change** | **95%CI** |
| --- | --- | --- |
| Orientation | -0.66 | -1.77 to -0.45 |
| Language | -1.25 | -2.06 to -0.61 |
| Memory | -0.12 | -0.81 to 0.64 |
| Attention | -0.31 | -0.83 to 0.22 |
| Praxis | -0.99 | -1.90 to -0.38 |
| Abstract | -0.43 | -1.81 to -0.90 |
| Thinking | -0.56 | -1.41 to -0.07 |
| Perception | 0.11 | -0.37 to 0.65 |
| Calculation | -0.27 | -0.43 to -0.12 |
