# Supplementary material for: Endothelium‐related biomarkers and cognitive decline in prevalent hemodialysis patients: A prospective cohort study
Source: Eur J Neurol. 2024 Aug 13;31(12):e16438. doi: 10.1111/ene.16438 (PMC11555003; doi:10.1111/ene.16438)
Supplement: Supplementary file 4 — Table S4. [file ENE-31-e16438-s002.docx]

**Supplementary Table S4:** Effect of baseline covariates on the slope (faster decline) of the CAMCOG score.

| **Risk Factor** | **Estimate (95% CI)** |
| --- | --- |
| Age, each 10 years | 0.15 (0.06 to 0.23) |
| Male gender | 0.15 (-0.09 to 0.40) |
| Years of education, each additional year | -0.01 (-0.02 to 0.002)* |
| Hypertension | -0.21 (-0.15 to 0.31) |
| Diabetes mellitus | 0.22 (-0.03 to 0.46) |
| Cardiovascular disease | -0.09 (-0.44 to 0.26) |
| Past or current smoking | 0.03 (-0.23 to 0.30) |
| Dialysis vintage, each 10 months | -0.03 (-0.22 to 0.16) |
| Single-pool K_t_/V, each 1 unit | -0.20 (-0.54 to 0.14) |
| Hemoglobin, each g/dL | -0.01 (-0.08 to 0.07) |
| Phosphorus, each mg/dL | 0.06 (-0.15 to 0.17) |
| Albumin, each g/dL | 0.18 (-0.09 to 0.45) |
| Parathormone, each 100 pg/mL | 0.21 (-0.15 to 0.14) |

*p=0.08
